# The Wheat Bax Inhibitor-1 Protein Interacts with an Aquaporin TaPIP1 and Enhances Disease Resistance in *Arabidopsis*

**DOI:** 10.3389/fpls.2018.00020

**Published:** 2018-01-22

**Authors:** Pan-Pan Lu, Tai-Fei Yu, Wei-Jun Zheng, Ming Chen, Yong-Bin Zhou, Jun Chen, You-Zhi Ma, Ya-Jun Xi, Zhao-Shi Xu

**Affiliations:** ^1^College of Agronomy, State Key Laboratory of Crop Stress Biology for Arid Areas, Northwest A&F University, Yangling, China; ^2^Chinese Academy of Agricultural Sciences, National Key Facility for Crop Gene Resources and Genetic Improvement, Key Laboratory of Biology and Genetic Improvement of Triticeae Crops, Ministry of Agriculture, Beijing, China

**Keywords:** Bax inhibitor-1, SA, *Pst* DC3000, biotic stress, TaPIP1, wheat

## Abstract

Bax inhibitor-1 (BI-1) is an endoplasmic reticulum (ER)-resident cell death suppressor evolutionarily conserved in eukaryotes. The ability of BI-1 to inhibit the biotic and abiotic stresses have been well-studied in *Arabidopsis*, while the functions of wheat BI-1 are largely unknown. In this study, the wheat BI-1 gene *TaBI-1.1* was isolated by an RNA-seq analysis of *Fusarium graminearum* (*Fg*)-treated wheat. *TaBI-1.1* expression was induced by a salicylic acid (SA) treatment and down-regulated by an abscisic acid (ABA) treatment. Based on β-glucuronidase (GUS) staining, *TaBI-1.1* was expressed in mature leaves and roots but not in the hypocotyl or young leaves. Constitutive expression of *TaBI-1.1* in *Arabidopsis* enhanced its resistance to *Pseudomonas syringae* pv. *Tomato* (*Pst*) DC3000 infection and induced SA-related gene expression. Additionally, *TaBI-1.1* transgenic *Arabidopsis* exhibited an alleviation of damage caused by high concentrations of SA and decreased the sensitivity to ABA. Consistent with the phenotype, the RNA-seq analysis of *35S::TaBI-1.1* and Col-0 plants showed that *TaBI-1.1* was involved in biotic stresses. These results suggested that *TaBI-1.1* positively regulates SA signals and plays important roles in the response to biotic stresses. In addition, TaBI-1.1 interacted with the aquaporin TaPIP1, and both them were localized to ER membrane. Furthermore, we demonstrated that *TaPIP1* was up-regulated by SA treatment and *TaPIP1* transgenic *Arabidopsis* enhanced the resistance to *Pst* DC3000 infection. Thus, the interaction between TaBI-1.1 and TaPIP1 on the ER membrane probably occurs in response to SA signals and defense response.

## Introduction

Crop plants are constantly exposed to a variety of biotic and abiotic stresses, such as drought, salinity and pathogen infection, leading to major losses in productivity. Unlike animals, plants cannot move to escape from environmental stresses. Plants have developed complex and elaborate mechanisms of induction and execute a wide range of physiological and metabolic responses to adapt to these stresses.

The plant hormone salicylic acid (SA) plays an important role in the plant response to biotic stress. As a key signal in the plant defense response, SA induces broad-spectrum systemic resistance by up-regulating the expression of pathogenesis-related (*PR*) genes and promoting cell wall rigidification and phytoalexin synthesis (Ryals et al., 1996; Dong, 1998; Pieterse and van Loon, 1999; Wildermuth et al., 2001). *PR1* is a well-known marker gene of SA-mediated disease resistance (Van Loon, 1998). The SA level is normally increased in tissues attacked by pathogens, which induces the expression of PR genes, including *PR1*, *PR2*, and *PR5*, to enhance plant resistance (Bari and Jones, 2008; Canet et al., 2012; Peng et al., 2012; Tateda et al., 2014; Choi et al., 2015). In addition, *ISOCHORISMATE SYNTHASE 1* (*ICS1*) and *DISEASE SUSCEPTIBILITY 1* (*EDS1*) also play key roles in disease resistance. *ICS1* encodes a key enzyme in SA biosynthesis (Wildermuth et al., 2001), whereas *EDS1* controls basal immunity by restricting the growth of virulent pathogens (Wiermer et al., 2005).

Salicylic acid synthesis is associated with the onset of the hypersensitive response (HR), which induces calcium influx, protein phosphorylation, dephosphorylation, the production of reactive oxygen intermediates, and *PR* gene expression. Extreme defense responses induce programmed cell death (PCD) (Morel and Dangl, 1997; Grant et al., 2000; Lam et al., 2001; Choi et al., 2012). PCD is crucial for defense responses to restrict the spread of pathogens in plants. Apoptotic-like PCD is one of the cell death pathways which results in a corpse morphology that is similar to the apoptotic morphology seen in animal cells (Reape and Mccabe, 2010). Bcl-2 family members are not present in fungi and reveal the pathways of apoptosis in animals (Breckenridge et al., 2003). Bcl-2 family proteins are divided into three subfamilies: Bcl-2 subfamily (pro-survival type; Bcl-2, Bcl-XL), Bax subfamily (pro-apoptotic type; Bax, Bak), and BH3 subfamily (pro-apoptotic type; Bad, Bid) (Ameisen, 2002; Watanabe and Lam, 2009). Unlike Bcl-2 family proteins, Bax inhibitor-1 (BI-1) is evolutionarily conserved and widely present in eukaryotic species from fungi to plants and animals (Chae et al., 2003). BI-1, as a highly conserved cell death suppressor, was identified based on its suppression of Bax-activated cell death in yeast (Xu and Reed, 1998; Kawai-Yamada and Uchimiya, 2001; Bolduc et al., 2003; Ishikawa et al., 2011; Lee et al., 2014). BI-1 is a cytoprotective protein localized to the endoplasmic reticulum (ER) membrane and plays important roles in responses to biotic and abiotic stresses (Watanabe and Lam, 2009). *AtBI-1*-overexpressing rice cells exhibit enhanced tolerance to menadione-induced oxidative stress and to infection with the rice blast fungus (*Magnaporthe grisea*) (Matsumura et al., 2003; Ishikawa et al., 2010) as well as an alleviation of stress-induced cell death; these effects are achieved by decreasing the levels of flotillin (FLOT) and HIR3, which are required for cell death induced by oxidative stress and SA (Ishikawa et al., 2015). AtBI-1 also suppresses cell death induced by pokeweed antiviral protein (PAP), Bax, H_2_O_2_, or SA (Kawai-Yamada et al., 2004). *AtBI-1* expression is induced by the non-phytopathogenic *hrpA* mutant of DC3000 and *Escherichia coli* (Sanchez et al., 2000). Overexpression of *HvBI-1* in single epidermal cells of barley enhances the resistance to the powdery mildew fungus *Blumeria graminis* f. sp. *Hordei* (Babaeizad et al., 2009). In *Arabidopsis*, *AtBI-1* plays a pivotal role in ER stress-mediated PCD. Two *AtBI-1* mutants (*atbi1-1* and *atbi1-2*) enhance sensitivity to the ER stress inducer tunicamycin (TM), and overexpression of *AtBI-1* markedly reduces the sensitivity to TM (Watanabe and Lam, 2008) compared with that of wild-type plants. The expression level of *Ss-Bi1* increases upon various stress treatments, and gene silenced lines are sensitive to heat stress and ER stress (Yu et al., 2015). Overexpression of *CaBI-1* in tobacco plants leads to a markedly improved tolerance to high temperature, water deficit, and high salinity (Isbat et al., 2009). The exogenous expression of wheat *TaBI-1* in tobacco partially blocks Bax-induced cell death caused, and the suppression of *TaBI-1* expression in wheat enhances the plant susceptibility to *Pst* (Wang et al., 2012).

To date, several proteins that interact with BI-1 have been identified. AtBI-1 interacts with calmodulin in yeast and plant cells and responds to ion homeostasis to regulate plant cell death (Ihara-Ohori et al., 2007). Human BI-1 interacts with NADPH-dependent cytochrome P450 oxidoreductase (NPR) and modulates ER stress-induced reactive oxygen species (ROS) accumulation (Hyung-Ryong et al., 2009). It have been demonstrated that BI-1 interacts with cytochrome b(5) and cytochrome P450 83A1 (CYP83A1) at the ER (Nagano et al., 2009; Weis et al., 2013). However, the knowledge about the mechanism of BI-1 in ER stress is also limited. Thus, a screen of candidate interacting proteins that are localized to the ER membrane may be an effective approach to better understand the function of BI-1 in ER stress.

Currently, the mechanisms by which BI-1 regulates the response to ER stress in plants are unclear, and the functions of BI-1 in responses to biotic and abiotic stresses are also poorly understood, requiring further investigation. Wheat is one of the most important cereals, providing ∼20% of the calories consumed by humans. Environmental stresses significantly suppress wheat growth and affect quality and yield. Therefore, studies aiming to understand the molecular mechanisms of genes involved in abiotic and biotic stress responses are necessary to enable the breeding of crops with stress tolerance. However, the function of wheat BI-1 is largely unknown. Here, we identified the wheat BI-1 gene *TaBI-1.1*, selected through an RNA-seq analysis of *Fusarium graminearum* (*Fg*)-treated wheat. TaBI-1.1 interacted with an aquaporin TaPIP1, and was co-localized with TaPIP1 on the ER membrane. Both *TaBI-1.1* and *TaPIP1* were SA-induced genes, and enhanced resistance to *Pseudomonas syringae* pv. *Tomato* (*Pst*) DC3000 in *Arabidopsis*, revealing a possible role of the interaction between TaBI-1.1 and TaPIP1 on the ER membrane in response to pathogen infection.

## Materials and Methods

### Plant Materials and Gene Cloning

*Arabidopsis* Columbia-0 (Col-0) was used as the background for overexpressing *TaBI-1.1*. The mutant *atbi1-2* (CS323793) was obtained from the *Arabidopsis* Biological Resource Center (ABRC). The cDNAs of *TaBI-1.1* (TRIAE_CS42_U_TGACv1_ 644608_AA2140670) and *TaPIP1* (TRIAE_CS42_6DL_TGACv1_526452_AA1684100) were amplified from the cultivated wheat cultivar Xiaobaimai (for primer sequences, see Supplementary Table S2). The PCR products were cloned into the pLB vector (TIANGEN, China). Amino acid sequence identity comparison was performed using the DNAMAN 6.0 software (Lynnon Biosoft, United States).

### Quantitative Real-Time PCR (qRT-PCR) Analysis

Wheat seedlings were grown at 22°C under a 16 h light/8 h dark photoperiod. 10-day-old wheat seedlings were used for SA, NaCl, and abscisic acid (ABA) treatments. For the SA, NaCl, and ABA treatments, wheat seedlings were sprayed with solutions containing 2 mM SA, 200 mM NaCl, and 1 μM ABA, respectively. For the NaCl and ABA treatments, samples were harvested at 0, 0.5, 1, 2, 4, 8, 12, and 24 h. For the SA treatment, the samples were harvested at 0, 1, 4, 8, 12, 24, 36, and 48 h. For the *Pst* DC3000 treatment, leaves of 4-week-old *Arabidopsis* seedlings were infiltrated with *Pst* DC3000 at OD600 = 0.002 and were collected at 0, 1, 2, and 3 days post-infection (dpi). For each time point, five leaves were collected from five individual plants. For the qRT-PCR analysis of SA-related genes, 2-week-old seedlings of Col-0, *atbi1-2*, and *35S::TaBI-1.1#1* grown under normal condition were used. All samples were rapidly frozen in liquid nitrogen and stored at -80°C. Three biological replicates were performed for different plants at each time point. RNA extraction and qRT-PCR were performed using the RNAprep plant kit (TIANGEN, China) and the ABI7500 real-time PCR system (ABI, United States), respectively. The specific primers and reference gene for the qRT-PCR were listed in Supplementary Table S2.

### Generation and Performance Evaluation of Transgenic *Arabidopsis* Plants under Stress Treatments

The coding sequences of *TaBI-1.1* and *TaPIP1* were cloned into the pCAMBIA1302 vector under the control of the CaMV 35S promoter using an In-Fusion HD Cloning Kit (Clontech, United States) (for primer sequences, see Supplementary Table S2). The fusions of *35S::TaBI-1.1* and *35S::TaPIP1* were transformed into Col-0 plants via the floral dipping method (Clough and Bent, 1998). The expression levels of *TaBI-1.1* and *TaPIP1* in transgenic *Arabidopsis* were detected using qRT-PCR, and sequenced to confirm transgenic plants constitutively overexpressing *TaBI-1.1* or *TaPIP1*. Homozygous T3 seeds of two transgenic lines of *TaBI-1.1* and *TaPIP1* with higher expression level were used for the further phenotypic analysis. The seeds were surface-sterilized, kept at 4°C for 3 days, and then sown on MS medium (Murashige and Skoog, 1962) supplemented with 2% Suc and solidified with 0.8% (w/v) agar at 22°C under long-day photoperiod (16 h light/8 h dark). For the phenotype analysis of *TaBI-1.1* transgenic plants, 4-day-old seedling plants per genotype were transferred to MS media containing 100 mM NaCl, 120 mM NaCl, 20 μM ABA, 30 μM ABA, 30 μM SA, and 50 μM SA for 13 days. The root length, fresh weight, and lateral root numbers of plants were monitored via statistical analysis. For the germination assay of *TaBI-1.1* transgenic plants, the seed number was recorded every 12 h post-incubation for visible radical emergence as a proxy for seed germination. Each treatment contained three independent replicates.

### β-Glucuronidase (GUS) Activity Assay

For the GUS activity assay, the promoter of *TaBI-1.1* was amplified from wheat cultivar Xiaobaimai and cloned into pCAMBIA1391Z using an In-Fusion HD Cloning Kit (Clontech, United States) (for primer sequences, see Supplementary Table S2) and transformed into Col-0 plants. The T2 generation transgenic seeds were germinated and grown on MS media for 5 days and then exposed to MS media containing 120 mM NaCl, 20 μM ABA, and 50 μM SA for 1 day under a day/night 16/8 h cycle at 22°C. Seedlings without any stress treatment were used as the control. GUS histochemical assays kit was used for detecting GUS activity (Real-Times, Beijing). The seedlings after staining were photographed using a Leica M165 FC stereomicroscope (Wetzlar, Germany).

### Bacterial Inoculation and Determination of Bacterial Growth

The *Pst* DC3000 was grown on King’s medium B (KB) (King et al., 1954) supplemented with 25 mg/L rifampicin for 48 h at 28°C and resuspended in 10 mM MgCl_2_ to OD_600_ = 0.002. Leaves of 4-week-old plants were infected with the bacterial suspension by pressing an 1-ml syringe (without a needle) against the abaxial side of the leaves and forcing the suspension through the stomata into the intercellular spaces. Each sample consisted of 20–25 infected leaves selected from five plants. Twenty leaves per genotype were harvested and photographed at 3 dpi. Eight leaf disks with 0.25 cm^2^ in size were cut from each genotype and ground into powder. Then, the materials were diluted with 10 mM MgCl_2_ and spread on KB medium. The medium was incubated for 2 days at 28°C. Eight replicate samples per genotype were assayed to obtain means and SD, which were determined from the logarithm of the number of c.f.u. cm^-2^.

### Yeast Two-Hybrid System

The MATCHMAKER two-hybrid system (Clontech, United States) was used for the yeast two-hybrid interaction assay. The coding regions of TaBI-1.1 and TaPIP1 were amplified and cloned into pGBKT7 and pGADT7 to create BD-TaBI-1.1, AD-TaBI-1.1, BD-TaPIP1, and AD-TaPIP1, respectively, using an In-Fusion HD Cloning Kit (Clontech, United States) (for primer sequences, see Supplementary Table S2). Seven groups, BD-TaBI-1.1 + AD-TaPIP1, AD-TaBI-1.1 + BD-TaPIP1, AD-TaBI-1.1 + BD, BD-TaBI-1.1 + AD, AD + BD-TaPIP1, BD + AD-TaPIP1 and AD + BD, were transformed into the yeast strain AH109 and selected by growing on SD/-Trp-Leu medium at 30°C for 4 days. Surviving clones were retransferred to SD/-Trp-Leu-His-Ade medium, according to the manufacturer instructions (Clontech, United States).

### Pull-Down assay

*TaBI-1.1* was cloned into the prokaryotic expression vector pCOLD (Takara, Japan), and *TaPIP1* was cloned into pGEX-4T-1 using an In-Fusion HD Cloning Kit (Clontech, United States) (for primer sequences, see Supplementary Table S2). GST pull-down assays was performed as described (Liu et al., 2013).

### Subcellular Localization Assay

The coding sequences without terminators of TaBI-1.1 and TaPIP1 were cloned into the p16318GFP vectors (for primer sequences, see Supplementary Table S2). For the subcellular localization analysis, TaBI-1.1-GFP and mRFP-HDEL, TaPIP1-GFP and mRFP-HDEL, TaBI-1.1-GFP and TaPIP1-mRFP were co-transformed into the wheat protoplasts via polyethylene glycol-mediated transformation (Marion et al., 2008). After 12 h of incubation in the dark at 22°C, the cells were observed using a confocal laser scanning microscope.

### ELISA Assay

Triplicate leaves with the weight of 0.5 g from 4-week-old seedlings were harvested and ground to fine powder in liquid nitrogen, and then, 450 μl methyl alcohol:PBS = 1:8 mixed liquor was added to the powder. The mixture was centrifuged at 12,000 rpm for 8 min at 4°C, and the supernatant was collected. For the ELISA assay, a Plant SA ELISA Kit (Rapidbio, United States) was used purified SA antibodies to coat microtiter plate wells to make solid-phase antibodies. Then, SA was added to wells, and combined SA antibodies were labeled with HRP to become an antibody-antigen-enzyme-antibody complex. After complete washing and TMB substrate solution addition, the TMB substrate became blue in HRP enzyme-catalyzed reactions. The reaction was terminated by the addition of a sulfuric acid solution, and the color change was measured spectrophotometrically at a 450 nm wavelength. The concentration of SA in the samples was determined by comparing the O.D. of the samples to the standard curve.

### RNA-Seq Analysis

At least 30 leaves from 4-week-old Col-0 and transgenic line *35S::TaBI-1.1* plants were collected for RNA-seq analysis (Allwegene, Beijing). The mRNA was isolated using poly-(T)-oligonucleotide-attached magnetic beads and fragmented to 100 to 200 bases. The double-strand cDNA was synthetized from mRNA using reverse transcriptase and random hexamer primers. Then, the cDNA fragments were purified using AMPure XP beads. Through an end-repair process, the addition of a single A base, and the ligation of the adapters, cDNA libraries were created via PCR enrichment. The libraries were sequenced using the HiSeq^TM^2500 sequencing system according to the manufacturer instructions (Illumina, United States). Sequencing reads were mapped to the TAIR 10 *Arabidopsis* reference genome using TopHat^1^ with default parameters. The abundance of assembled transcripts was calculated in fragments per kilobase of exon model per million mapped fragments (FPKM). The TopHat and Cufflink software packages were used for the mRNAseq data analysis to identify DEGs. For the no-biological-repeat RNA-seq analysis, the readcount data needed to be standardized using TMM, and the threshold value of differentially expressed genes was | log2 (FoldChange)| > 1 and *P*-Adjusted < 0.005. The hierarchical clustering analysis was generated via the RPKM of differentially expressed genes of *35S::TaBI-1.1* VS Col-0. The GO terms enrichment of differentially expressed genes was conducted using the GOseq software based on the Wallenius non-central hypergeometric distribution. The top 30 enriched GO terms were shown in histograms for up-regulated and down-regulated genes. The KEGG enrichment analysis, based on pathways from the KEGG database, used a hypergeometric examination to find the enriched pathways in differentially expressed genes compared with the transcriptome background. The wheat *Fg* treated RNA-seq data was download from SRA database (accession number: PRJNA289545) and analyzed by BMKCloud (BIOMARKER, China).

## Results

### The Expression Patterns of *TaBI-1.1* Were Determined via qRT-PCR and β-Glucuronidase (GUS) Staining

**FIGURE 1:**
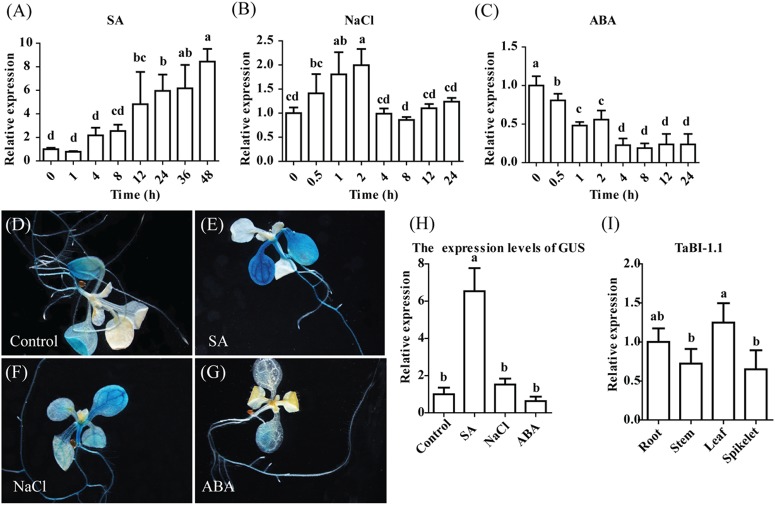
Relative and spatial expression patterns of *TaBI-1.1* were evaluated by qRT-PCR and GUS staining. qRT-PCR analysis showing relative expression of *TaBI-1.1* in the wheat seedlings with SA **(A)**, NaCl **(B)**, and ABA **(C)** treatments. The vertical coordinates represent fold changes, and the horizontal coordinates represent time. Wheat *actin* was used as a reference. **(D–G)** GUS staining for *TaBI-1.1* expression in seedlings grown in the presence of SA, NaCl and ABA treatments. Seedlings grown under normal condition were used as the control. **(H)** qRT-PCR analysis of GUS expression levels in the PBI:GUS transgenic lines. The vertical coordinates represent fold changes, and the horizontal coordinates represent different treatments. *Arabidopsis actin2* was used as a reference. **(I)** qRT-PCR analysis of the transcript levels of *TaBI-1.1* in roots, stems, leaves, and spikelets wheat tissues. The vertical coordinates represent fold changes, and the horizontal coordinates represent different tissues. Wheat *actin* was used as a reference. Three biological replicates were taken for qRT-PCR. Error bars indicate standard deviations (SDs). All the data represent the means ± SDs of three independent biological replicates. Different letters in bar graphs indicate significant differences.

We analyzed the RNA-seq data from *Fg*-treated wheat to investigate the plant defense signaling pathway. Using the transcript count threshold for cytokinin (CK) of greater than 100 (as the control for *Fg* treatment, “CK1_Count,” “CK2_Count,” and “CK3_Count” represent three replicates) and the fragments per kilobase of transcript per million mapped reads (FPKM) threshold for CK of greater than 15 (“CK1_FPKM,” “CK1_FPKM,” and “CK1_FPKM” are three replicates), we isolated the top 10 differentially expressed genes from the RNA-seq analysis (Supplementary Table S1). Two wheat BI-1 genes (TRIAE_CS42_U_TGACv1_644608_AA2140670 and TRIAE_CS42_6BS_TGACv1_515717_AA1671980) were identified among the 10 genes. TRIAE_CS42_6BS_TGACv1_515717_AA1671980 was named *TaBI-1* in a previous study (Wang et al., 2012). Only three BI-1 genes were screened in the wheat Ensembl database: *TaBI-1*, TRIAE_CS42_U_TGACv1_644608_AA2140670 and TRIAE_CS42_6AS_TGACv1_488014_AA1573990. Of the two differentially expressed BI-1 genes, TRIAE_CS42_U_TGACv1_644608_AA2140670, which was named *TaBI-1.1*, displayed the greatest difference in expression. TaBI-1.1 shared 99.19% identity with TaBI-1, according to the amino acid alignment. The expression level of *TaBI-1.1* was up-regulated 24-fold in response to the *Fg* treatment compared with that in CK. Many studies on *AtBI-1* have confirmed that *AtBI-1* plays a pivotal role in plant resistance to biotic and abiotic stresses. The sequence of the TaBI-1.1 protein shared 69.88% identity with AtBI-1, indicating substantial conservation of this sequence in the BI-1 family (Sanchez et al., 2000). The expression patterns of *TaBI-1.1* were monitored using qRT-PCR to determine the possible role of *TaBI-1.1* in biotic and abiotic stresses. *TaBI-1.1* expression was significantly up-regulated in response to the SA treatment and down-regulated in response to the ABA treatment. *TaBI-1.1* expression peaked at ∼8-fold at 48 h after the SA treatment (**Figure 1A**). The level of down-regulation in response to the ABA treatment reached ∼1/5 of the initial level at 8 h (**Figure 1C**). In response to the NaCl treatment, the expression level declined at 4 h after a slight increase at 2 h and returned to its initial level at 8 h (**Figure 1B**).

We created a PBI::GUS fusion construct that contained 1.7 kb of the *TaBI-1.1* promoter and generated transgenic *Arabidopsis* to investigate the spatial expression pattern of *TaBI-1.1*. The histological GUS activity was determined using GUS staining. *TaBI-1.1* expression was observed in mature leaves and roots, but not in hypocotyl and young leaves (**Figure 1D**). We also examined the *TaBI-1.1* expression levels in various wheat tissues: roots, stems, leaves, and spikelets, and the results showed that the expression of *TaBI-1.1* was ubiquitous in these wheat tissues, and it was highest in leaves and lowest in spikelets (**Figure 1I**). Following the SA and NaCl treatments, the expression in mature leaves was increased compared with that in the control, and higher expression was observed in response to the SA treatment. Expression was also detected in the hypocotyl upon exposure to SA and NaCl treatments (**Figures 1E,F**). Weaker expression was detected in the leaves of ABA-treated plants (**Figure 1G**). GUS expression levels were further confirmed by qRT-PCR (**Figure 1H**). Based on these results, *TaBI-1.1* expression responded to various stresses.

### Constitutive Expression of *TaBI-1.1* Enhanced Resistance to *Pst* DC3000 Infection in *Arabidopsis*

**FIGURE 2:**
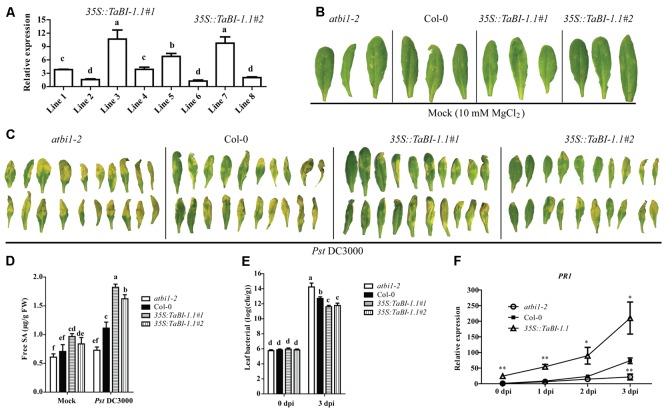
*TaBI-1.1* transgenic *Arabidopsis* exhibited enhanced resistance to *Pst* DC3000. **(A)** qRT-PCR analysis of *TaBI-1.1* transcripts in eight *35S::TaBI-1.1* transgenic lines. The line 6 with the lowest expression levels was set to “1.” *Arabidopsis actin2* was used as a reference. Bacterial defense phenotypes on 4-week-old *atbi1-2*, Col-0, *35S::TaBI-1.1#1*, and *35S::TaBI-1.1#2* plant leaves exposed to 10 mM MgCl_2_ (mock) **(B)** and *Pst* DC3000 (OD_600_ = 0.002) **(C)** observed at 3 dpi. **(D)** Free SA levels in *atbi1-2*, Col-0, *35S::TaBI-1.1#1*, and *35S::TaBI-1.1#2* leaves exposed to 10 mM MgCl_2_ (mock) and *Pst* DC3000 (OD_600_ = 0.002) after 24 h. Different letters in bar graphs indicate significant differences. **(E)** The bacterial titre (log10) of *atbi1-2*, Col-0, *35S::TaBI-1.1#1*, and *35S::TaBI-1.1#2* leaves at 0 and 3 dpi with *Pst* DC3000 (OD_600_ = 0.002). Different letters in bar graphs indicate significant differences. **(F)** qRT-PCR analysis showing the *PR1* expression levels at 0, 1, 2, and 3 dpi with *Pst* DC3000 (OD_600_ = 0.002). The expression level of *PR1* in Col-0 at 0 dpi was set to “1.” *Arabidopsis actin2* was used as a reference. Asterisks indicate significant differences (^∗^*P* < 0.05 and ^∗∗^*P* < 0.01) compared with Col-0 (Student’s *t*-test). Error bars indicate SDs. All the data represent the means ± SDs of three independent biological replicates.

Based on its up-regulation in response to *Fg* and SA treatments, we hypothesized that *TaBI-1.1* may be involved in responses to biotic stresses. We ectopically expressed *TaBI-1.1* in *Arabidopsis* under the control of the cauliflower mosaic virus (CaMV) 35S promoter to verify this hypothesis. Two homozygous lines with relatively high *TaBI-1.1* expression levels, lines 3 and 7 (*35S::TaBI-1.1#1* and *35S::TaBI-1.1#2*), were selected for further analysis (**Figure 2A**). Four-week-old leaves of *atbi1-2*, Col-0, and two transgenic lines were subjected to a *Pst* DC3000 infection or 10 mM MgCl_2_ (mock). Under the mock treatment, no obviously differences were observed among leaves from *atbi1-2*, Col-0, and the two transgenic lines 3 days after treatment with 10 mM MgCl_2_ (**Figure 2B**). Disease symptoms were detected in these leaves 3 days after inoculation with *Pst* DC3000 at an optical density at 600 nm (OD_600_) of 0.002. The *atbi1-2* mutant clearly exhibited severe symptoms, as almost all leaves showed serious chlorosis and necrosis, whereas the two transgenic lines showed milder disease symptoms than *atbi1-2* and Col-0. A small portion of leaves of transgenic plants were green and did not display chlorosis and necrosis. The degree of the disease symptoms in Col-0 leaves was intermediate between *atbi1-2* and the two transgenic lines (**Figure 2C**). The SA level was monitored at 24 h after inoculation with *Pst* DC3000 or 10 mM MgCl_2_ (mock). In the mock group, the highest SA level was observed in *35S::TaBI-1.1#1*, which was significantly higher than that in Col-0. Under *Pst* DC3000 treatment, significantly higher SA levels were detected in the two transgenic lines compared with Col-0, and *35S::TaBI-1.1#1* contained the highest SA level among the four genotypes. The difference between Col-0 and *atbi1-2* also reached a significant level (**Figure 2D**). The bacterial titres in leaves of *atbi1-2*, Col-0, and the two transgenic lines were measured at 0 and 3 dpi. No significant differences in pathogenic bacterial growth were observed among the four genotypes in the initial inoculation amount (0 dpi). At 3 dpi, the bacterial titres of the two transgenic lines were significantly lower than those of Col-0, and the bacterial titre of Col-0 was also significantly lower than that of *atbi1-2*, indicating that the two transgenic lines exhibited a substantial inhibition of pathogenic bacterial growth, and that *atbi1-2* was more susceptible to pathogenic bacteria than Col-0 (**Figure 2E**). Based on the higher level of *35S::TaBI-1.1#1* in two transgenic lines, we used *35S::TaBI-1.1#1* to further detect the expression of *PR1*. High SA levels induce the expression of *PR* genes to enhance plant resistance to pathogen attack (Ohshima et al., 1990). The increase in *PR1* expression may explain the enhancement of the resistance to *Pst* infections (**Figure 2F**).

### *TaBI-1.1* Positively Regulated SA-Related Gene Expression

**FIGURE 3:**
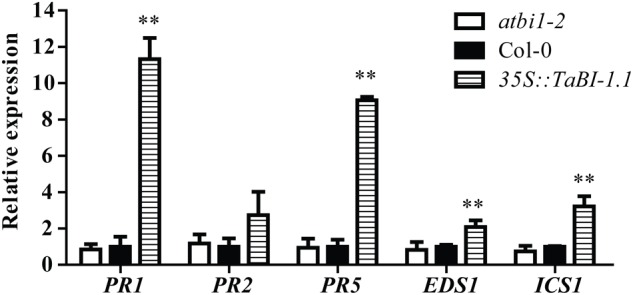
The expression levels of five SA-related genes in 2-week-old seedlings of *atbi1-2*, Col-0, and *35S::TaBI-1.1* transgenic *Arabidopsis* grown under normal condition were monitored by qRT-PCR. *Arabidopsis actin2* was used as a reference. The expression levels of genes in Col-0 were set to “1.” *Arabidopsis actin2* was used as a reference. Error bars indicate the SDs. The data represent the means ± SDs of three independent replicates. Asterisks indicate significant differences (^∗^*P* < 0.05 and ^∗∗^*P* < 0.01) compared with Col-0 (Student’s *t*-test).

*PR* gene expression is associated with SA accumulation and systemic acquired resistance (SAR) (Wildermuth et al., 2001). We further examined SA-related genes expression in 2-week-old seedlings of Col-0, *atbi1-2*, and *35S::TaBI-1.1#1* grown under normal condition. Significantly higher expression levels of *PR1*, *PR5*, *ICS1*, and *EDS1* were observed in *35S::TaBI-1.1* than in Col-0 and *atbi1-2*. However, no significant difference was detected between Col-0 and *atbi1-2* in these genes expression levels (**Figure 3**). The expression levels of *PR1* and *PR5* in *35S::TaBI-1.1* increased ∼11- and ∼9-fold, respectively, compared with those in Col-0. The fold changes in *PR2*, *ICS1* and *EDS1* expression were lower than the changes in *PR1* and *PR5* expression (**Figure 3**). Thus, *TaBI-1.1* up-regulated the expression of the *PR1*, *PR2*, *PR5*, *ICS1* and *EDS1* genes, indicating a positive role of *TaBI-1.1* in SA signaling.

### *TaBI-1.1* Decreased the Sensitivity to SA and ABA

**FIGURE 4:**
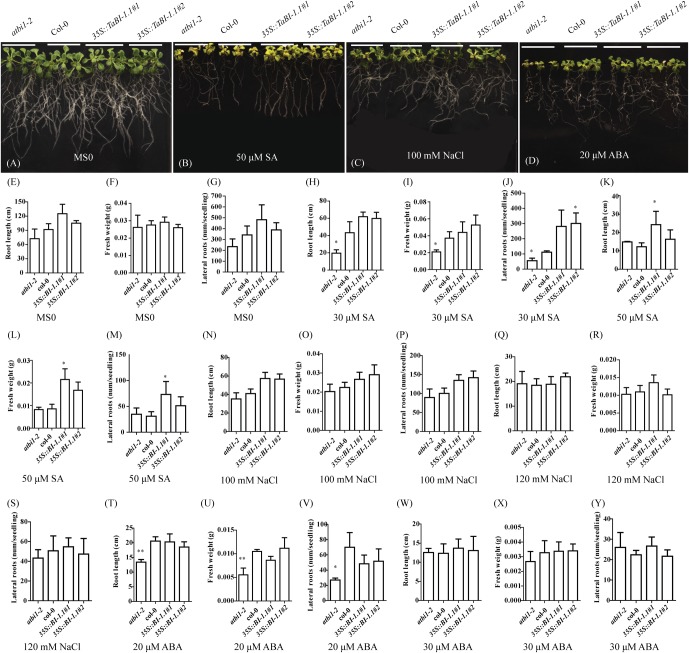
Statistical analysis of seedling root length, fresh weight, and lateral root numbers of *atbi1-2*, Col-0, and *35S::TaBI-1.1* transgenic *Arabidopsis* grown on MS media containing SA, NaCl, or ABA. Growth phenotype of *atbi1-2*, Col-0, and the two *35S::TaBI-1.1* transgenic seedlings on **(A)** MS0 medium, **(B)** MS medium containing 50 μM SA, **(C)** MS medium containing 100 mM NaCl, and **(D)** MS medium containing 20 μM ABA. After stratification, seedlings were grown in MS0 medium for 4 days in a horizontal position and then transported to MS media containing different concentrations of SA, NaCl, and ABA and grown for 13 days in a vertical position. These experiments were repeated with nine biological replicates, and the photographs show three representative seedlings of four genotypes. Statistical analysis of the root length of *atbi1-2*, Col-0, and the two *35S::TaBI-1.1* transgenic lines on MS0 **(E)**, MS media containing 30 μM SA **(H)**, 50 μM SA **(K)**, 100 mM NaCl **(N)**, 120 mM NaCl **(Q)**, 20 μM ABA **(T)**, and 30 μM ABA **(W)**. Statistical analysis of the fresh weight of *atbi1-2*, Col-0, and the two *35S::TaBI-1.1* transgenic lines on MS0 **(F)**, MS media containing 30 μM SA **(I)**, 50 μM SA **(L)**, 100 mM NaCl **(O)**, 120 mM NaCl **(R)**, 20 μM ABA **(U)**, and 30 μM ABA **(X)**. Statistical analysis of the root length of *atbi1-2*, Col-0, and the two *35S::TaBI-1.1* transgenic lines on MS0 **(G)**, MS media containing 30 μM SA **(J),** 50 μM SA **(M)**, 100 mM NaCl **(P)**, 120 mM NaCl **(S)**, 20 μM ABA **(V)**, and 30 μM ABA **(Y)**. Error bars indicate the SDs. The data represent the means ± SDs of nine independent biological replicates. Asterisks indicate significant differences (^∗^*P* < 0.05 and ^∗∗^*P* < 0.01) compared with Col-0 (Student’s *t*-test).

Four-day-old seedlings of Col-0, *atbi1-2*, and *35S::TaBI-1.1* were placed in Murashige and Skoog (MS) medium containing different concentrations of SA, NaCl, and ABA to investigate the different phenotypes among Col-0, *atbi1-2*, and *35S::TaBI-1.1* transgenic *Arabidopsis* plants at the seedling stage in response to SA and other stress treatments. The morphological changes were monitored after 13 days. On MS medium without growth regulators (MS0), no significant differences in root length, lateral roots, or fresh weight were observed between any two genotypes of Col-0, *atbi1-2*, and two transgenic lines of *35S::TaBI-1.1* (**Figures 4A,E–G**). On MS medium supplemented with 30 μM SA, *atbi1-2* exhibited significant differences from Col-0 in fresh weight, root length and lateral root numbers (**Figures 4H–J**). The lateral root numbers of *35S::TaBI-1.1#2* were also significantly different from those of Col-0 (**Figure 4J**). The degree of aberrant growth of Col-0 was intermediate between *atbi1-2* and *35S::TaBI-1.1* plants grown on MS medium containing 30 μM SA (**Figures 4H–J**). High SA concentrations increase the accumulation of H_2_O_2_ and lead to oxidative damage (Horváth et al., 2007). When placed on MS medium containing 50 μM SA, seedlings showed more evident growth malformation. Based on these results, seedlings were subjected to more severe SA stress in the presence of higher SA concentrations. Leaves that had been treated with a high concentration of SA were pale green, yellowish and smaller than leaves treated with MS0 medium (**Figure 4B**). The root length of *35S::TaBI-1.1#1* grown on MS medium containing 50 μM SA was significantly longer than that of Col-0 (**Figure 4K**). The fresh weight and lateral root number of *35S::TaBI-1.1#1* were significantly increased compared with those of Col-0 (**Figures 4L,M**). Thus, SA not only affected the size and color of leaves but also the root length, fresh weight, and lateral root number.

Additionally, we examined the phenotype of seedlings grown on NaCl- and ABA-containing MS medium. No significant differences were observed among plants grown on 100 and 120 mM NaCl-containing MS medium (**Figures 4C,N–S**). Following treatment with 20 μM ABA, the degree of malformation of *atbi1-2* was clearly much more severe than that of Col-0 and *35S::TaBI-1.1* plants (**Figure 4D**). According to the statistical analyses, *atbi1-2* exhibited significant differences from Col-0 in root length, fresh weight, and lateral root number, indicating greater sensitivity to ABA (**Figures 4T–V**). However, no obvious differences in root length, fresh weight, or lateral root numbers were observed in response to the 30 μM ABA treatment (**Figures 4W–Y**). Thus, *TaBI-1.1* transgenic *Arabidopsis* exhibited decreased sensitivity to high concentrations of SA, and the *atbi1-2* mutant exhibited increased sensitivity to ABA.

**FIGURE 5:**
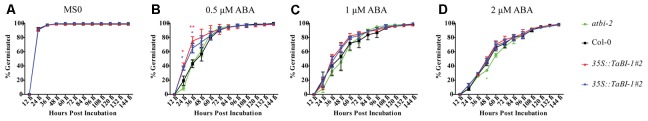
*TaBI-1.1* transgenic *Arabidopsis* exhibit decreased sensitivity to ABA during germination. Germination of *atbi1-2*, Col-0, and *35S::TaBI-1.1* transgenic *Arabidopsis* on MS0 medium **(A)** or medium containing 0.5 μM ABA **(B)**, 1 μM ABA **(C)**, or 2 μM ABA **(D)**. The vertical coordinates represent the percentages of germinated seeds, and the horizontal coordinates represent hours post-incubation. The number of germinated seeds was recorded every 12 h post-incubation. Error bars indicate the SDs. The data represent the means ± SDs of three independent biological replicates. Asterisks indicate significant differences (^∗^*P* < 0.05 and ^∗∗^*P* < 0.01) compared with Col-0 (Student’s *t*-test).

We further studied the sensitivity of *TaBI-1.1* to ABA during germination. The percentage of germinated seeds was recorded every 12 h on medium with different concentrations of ABA. On MS0 medium, Col-0, *atbi1-2*, and *35S::TaBI-1.1* transgenic *Arabidopsis* plants showed similar germination rates (**Figure 5A**). On medium containing 0.5 μM ABA, the two transgenic lines germinated significantly faster than Col-0 and *atbi1-2* (**Figure 5B**). No significant difference was observed on medium containing 1 μM ABA (**Figure 5C**). On medium containing 2 μM ABA, Col-0 and the two transgenic lines also germinated slightly faster than *atbi1-2* (**Figure 5D**). Based on these results, *TaBI-1.1* transgenic *Arabidopsis* exhibited decreased sensitivity to ABA.

### RNA-Seq Analysis of the Constitutive Expression of *TaBI-1.1* in *Arabidopsis*

**FIGURE 6:**
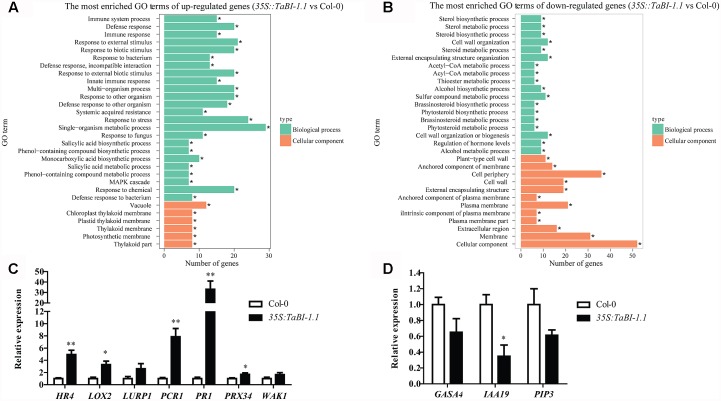
The enrichment of GO terms for genes that were differentially expressed between *35S::TaBI-1.1* and Col-0 was analyzed using RNA-seq. The most enriched GO terms of up-regulated genes **(A)** and down-regulated genes **(B)** in *35S::TaBI-1.1* compared with Col-0 (*35S::TaBI-1.1* vs. Col-0). Vertical coordinates represent enriched GO terms, and horizontal coordinates represent the numbers of differentially expressed genes for these GO terms. The green columns represent the GO terms for biological processes, and the orange columns represent the GO terms for cellular components. The asterisk “^∗^” indicates significantly enriched GO terms. **(C)** qRT-PCR analysis of seven up-regulated genes in *35S::TaBI-1.1* vs. Col-0. **(D)** qRT-PCR analysis of three down-regulated genes in *35S::TaBI-1.1* vs. Col-0. Vertical coordinates represent fold changes, and horizontal coordinates represent different genes. The relative expression levels of 10 genes in Col-0 were set to “1.” *Arabidopsis actin2* was used as a reference. Error bars indicate the SDs. The data represent the means ± SD of three biological replications. Asterisks (^∗^ and ^∗∗^) indicate significant differences (*P* < 0.05 and *P* < 0.01) compared with Col-0 (Student’s *t*-test).

We conducted an RNA-seq analysis of *35S::TaBI-1.1#1* and Col-0 plants to obtain a better understanding of *TaBI-1.1* function. Forty-eight up-regulated genes and 58 down-regulated genes were identified and used to create a heat map (Supplementary Figure S1). Based on the fold change [false discovery rate (FDR) ≤ 0.05], differentially expressed genes were categorized into functional groups using Gene Ontology (GO) analyses. More than 30 functionally enriched GO terms were identified for up-regulated and down-regulated genes, and the top 30 enriched GO terms were shown in **Figure 6**. For up-regulated genes, the distribution of enriched GO terms focused on biological processes related to resistance to biotic stresses, the cellular immune response, and SA synthesis and metabolism. The top 10 key GO terms were defense response, response to an external stimulus, response to a biotic stimulus, response to an external biotic stimulus, multi-organism process, response to another organism, defense response to another organism, response to stress, single-organism metabolic process, and response to chemicals (**Figure 6A**). For down-regulated genes, the distribution of enriched GO terms focused on the cellular components, and the top three terms were cellular components, membrane, and cell periphery (**Figure 6B**). No substantially enriched GO terms were related to biological processes. Seven up-regulated genes and three down-regulated genes were selected for qRT-PCR experiments to verify the accuracy of the RNA-seq analysis. These genes include *PLANT CADMIUM RESISTANCE 1* (*PCR1*), *CELL WALL-ASSOCIATED KINASE 1* (*WAK1*), *LATE UPREGULATED IN RESPONSE TO HYALOPERONOSPORA PARASITICA* (*LURP1*), *PR1*, *LIPOXYGENASE 2* (*LOX2*), *PEROXIDASE 34* (*PRX34*), *HOMOLOG OF RPW8 4* (*HR4*), *INDOLE-3-ACETIC ACID INDUCIBLE 19* (*IAA19*), *GAST1 PROTEIN HOMOLOG 4* (*GASA4*), and *PLASMA MEMBRANE INTRINSIC PROTEIN 3* (*PIP3*). The qRT-PCR results were consistent with the RNA-seq analysis. Higher expression levels of *PCR1*, *WAK1*, *LURP1*, *PR1*, *LOX2*, *PRX34*, and *HR4* were detected in *35S::TaBI-1.1* plants than in Col-0 and were increased by 4.9-, 3.3-, 2.6-, 7.9-, 33-, 1.7- and 1.7-fold, respectively (**Figure 6C**). Lower expression levels of *GASA4*, *IAA19*, and *PIP3* were detected in *35S::TaBI-1.1* plants than in Col-0, reaching 0.7-, 0.3- and 0.6-fold, respectively (**Figure 6D**).

**FIGURE 7:**
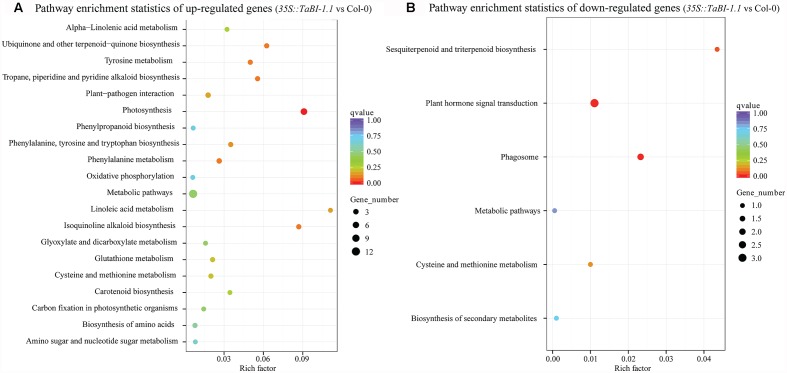
KEGG enrichment analysis of differentially expressed genes between *35S::TaBI-1.1* and Col-0. The pathway enrichment statistics of up-regulated genes **(A)** and down-regulated genes **(B)** in *35S::TaBI-1.1* vs. Col-0. Vertical coordinates represent enriched pathways, and horizontal coordinates represent enrichment factors. The size of each point represents the number of differentially expressed genes in the pathway, and the color of the point represents the *q*-value.

A Kyoto Encyclopaedia of Genes and Genomes (KEGG) enrichment analysis of differentially expressed genes was presented as a scatter diagram. The enrichment factor, *q*-value, and the number of genes that were enriched in a pathway were used to measure the degree of enrichment. A greater enrichment factor represents a higher degree of enrichment. More than 20 KEGG-enriched pathways were observed for up-regulated genes. The top 20 enriched pathways for the up-regulated genes were shown, with photosynthesis as the most obvious enriched pathway (**Figure 7A**). The enrichment factor for photosynthesis reached 0.09, and the *q*-value was approximately 0. In contrast, the KEGG-enriched pathways for the down-regulated genes were not as obvious, and only six pathways with lower enrichment factors were identified in the KEGG enrichment analysis (**Figure 7B**). Thus, *TaBI-1.1* was involved in responses to biotic stresses and performed its defense function mainly by up-regulating gene expression.

### TaBI-1.1 Interacted with TaPIP1 and Was Co-localized with TaPIP1 at the ER Membrane

*TaBI-1.1* was placed in the PGBKT7 (BD) vector and used as the bait protein to screen a wheat cDNA library in the yeast two-hybrid assay to further explore the cellular mechanisms by which TaBI-1.1 regulates the stress response. As a result, one candidate interacting partner was identified, the aquaporin TaPIP1. Yeast two-hybrid and pull-down assays were used to determine the interaction between TaBI-1.1 and TaPIP1 *in vivo* and *in vitro*.

**FIGURE 8:**
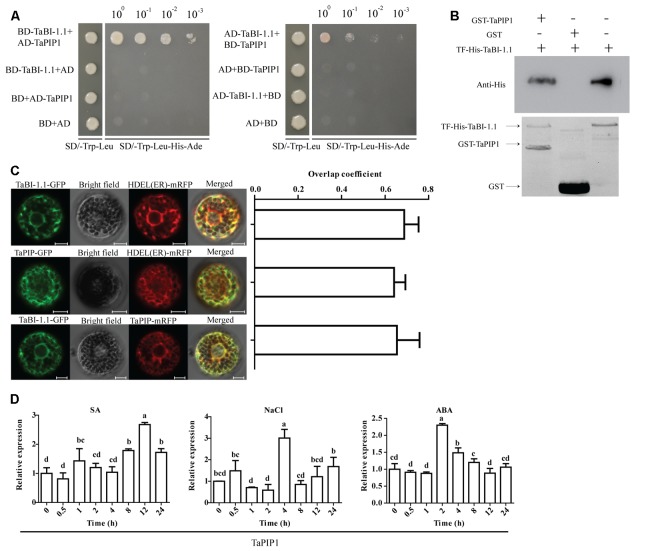
The interaction and subcellular localization of TaBI-1.1 and TaPIP1. **(A)** The interaction between TaBI-1.1 and TaPIP1 was measured using the yeast two-hybrid assay. Seven vector combinations were co-transformed into the yeast AH109 strain, and transformants were placed on SD/-Trp-Leu-His-Ade medium and grown for 4 days. **(B)** The interaction between TaBI-1.1 and TaPIP1 was measured using a pull-down assay. A western blot with an anti-His antibody was used to detect interactions, and the interaction between TF-His-TaBI-1.1 and GST was used as a control. **(C)** Co-localizations between TaBI-1.1-GFP and mRFP-HDEL, TaPIP1-GFP and mRFP-HDEL, TaBI-1.1-GFP and TaPIP1- mRFP in wheat protoplasts. The left panel shows the fluorescence of GFP, mRFP and the merged image. The right panel shows the levels of co-localization calculated from overlap coefficients obtained from at least 10 individual protoplasts. **(D)** Expression profiles of *TaPIP1* after SA, NaCl, and ABA treatments for 0, 0.5, 1, 2, 4, 8, 12, and 24 h. Wheat *actin* was used as a reference. Vertical coordinates represent fold changes, and horizontal coordinates represent different time periods. Error bars indicate the SDs. The results represent the means ± SDs of three biological replicates. Different letters in bar graphs indicate significant differences.

A BD vector fusing TaBI-1.1 (BD-TaBI-1.1) and a pGADT7 (AD) vector fusing TaPIP1 (AD-TaPIP1) were transformed into yeast cells. Only the yeast cells transformed with BD-TaBI-1.1 and AD-TaPIP1 were able to grow on a selective medium that lacked Trp, Leu, His, and Ade (SD/-Trp-Leu-Ade-His). In contrast, the transformants expressing BD-TaBI-1.1 and AD, BD and AD-TaPIP1 or BD and AD did not grow on the SD/-Trp-Leu-Ade-His medium. Switching the fusion vectors of TaBI-1.1 and TaPIP1 yielded the same results, indicating that TaBI-1.1 interacted with TaPIP1 in yeast cells (**Figure 8A**). The interaction was further confirmed using a pull-down assay (**Figure 8B**). *TaBI-1.1* was cloned into the pCold^TM^ TF expression vector to produce the recombinant protein TF-His-TaBI-1.1 in *E. coli*. The recombinant GST (glutathione *S*-transferase)-TaPIP1 protein was successfully produced in *E. coli* by cloning the sequence into the pGEX-4T-1 vector. *In vitro* pull-down assays showed that TF-His-TaBI-1.1 physically interacted with GST-TaPIP1, as shown in western blots using anti-His antibodies (**Figure 8B**).

AtBI-1 is localized to the ER membrane (Watanabe and Lam, 2009). We detected the co-localization of TaBI-1.1-GFP and mRFP-HDEL (an ER marker) in wheat protoplasts to determine the subcellular localization of TaBI-1.1 (Gomord et al., 1997). The overlap coefficient of GFP and mRFP fluorescence was 0.69, indicating that TaBI-1.1 was co-localized with HDEL at the ER membrane (**Figure 8C**). In view of the interaction between TaBI-1.1 and TaPIP1, we also detected co-localization between TaPIP1-GFP and mRFP-HDEL and co-localization between TaBI-1.1-GFP and TaPIP1-mRFP in wheat protoplasts (**Figure 8C**). The results showed that TaBI-1.1 and TaPIP1 were co-localized to the ER membrane.

The expression levels of *TaPIP1* in response to multiple treatments were monitored by qRT-PCR. *TaPIP1* expression was up-regulated by SA, NaCl and ABA treatments, reaching peak expression levels of ∼2.7-fold at 12 h, ∼3-fold at 4 h and ∼2.3-fold at 2 h, respectively, implying that TaPIP1 was probably involved in responses to biotic and abiotic stresses (**Figure 8D**).

### *TaPIP1* Increased the Resistance to *Pst* DC3000 Infection in *Arabidopsis*

**FIGURE 9:**
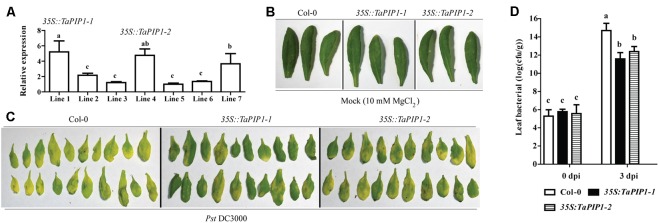
*TaPIP1* transgenic *Arabidopsis* exhibited enhanced resistance to *Pst* DC3000. **(A)** qRT-PCR analysis of *TaPIP1* transcripts in seven *35S::TaPIP1* transgenic lines. The line 5 with the lowest expression level was set to “1.” Bacterial defense phenotypes on 4-week-old Col-0, *35S::TaPIP1-1*, and *35S::TaPIP1-2* plant leaves exposed to 10 mM MgCl_2_ (mock) **(B)** and *Pst* DC3000 (OD_600_ = 0.002) **(C)** observed at 3 dpi. **(D)** The bacterial titre (log10) of Col-0, *35S::TaPIP1-1*, and *35S::TaPIP1-2* leaves at 0 and 3 dpi with *Pst* DC3000 (OD_600_ = 0.002). *Arabidopsis actin2* was used as a reference. Error bars indicate SDs. The data represent the means ± SDs of three independent biological replicates. Different letters in bar graphs indicate significant differences.

To further investigate the potential functions of the interaction between TaBI-1.1 and TaPIP1, we generated the transgenic *Arabidopsis* that constitutively expressing *TaPIP1* under the control of the CaMV 35S promoter. We selected two independent transgenic lines, lines 1 and 4 (*35S::TaPIP1-1* and *35S::TaPIP1-2*), with higher expression levels of *TaPIP1* as confirmed by transcript analysis using qRT-PCR (**Figure 9A**). Based on the amino acids sequence analysis, AtPIP1;4 shares the highest identity with TaPIP1 in *Arabidopsis* and is required for cytoplasmic import of apoplastic H_2_O_2_ induced by the bacterial pathogen (Tian et al., 2016). In view of the up-regulation of *TaPIP1* levels under SA treatment, we speculated that *TaPIP1* may be also involved in response to pathogen infection. To test this idea, we examined the leaves phenotypes of *TaPIP1* transgenic *Arabidopsis* under *Pst* DC3000 infection. Under the mock treatment, no differences were observed among leaves from Col-0 and the two transgenic lines (**Figure 9B**). After inoculation with *Pst* DC3000, we found obvious chlorotic symptoms on the leaves of Col-0, in contrast, chlorotic symptoms of two transgenic plants were milder (**Figure 9C**). To further confirm the increased resistance in *TaPIP1* transgenic *Arabidopsis*, we measured the bacterial titer in leaves at 0 and 3 dpi. As shown in **Figure 9D**, there were no significant differences among Col-0 and the two transgenic lines in the initial inoculation amount, however, the bacterial titer was significantly lower in two *TaPIP1* transgenic *Arabidopsis* compared with Col-0 at 3 dpi, indicating that pathogenic bacterial growth was greatly inhibited in *TaPIP1* transgenic *Arabidopsis*. Therefore, *TaBI-1.1* and *TaPIP1* exhibited similar roles in response to *Pst* DC3000 infection, and we speculated that the interaction between *TaBI-1.1* and *TaPIP1* was probably involved in defense response.

## Discussion

Salicylic acid is a key signal in disease resistance, inducing local acquired resistance (LAR) and SAR, and leads to the increased expression of many defense proteins, including PR proteins. The expression of *PR* genes is positively correlated with SA accumulation and pathogen resistance. *PR1* is one of the most important marker genes in SA-mediated disease resistance. In this study, *TaBI-1.1* transgenic *Arabidopsis* showed a higher SA level, higher *PR1* expression level, and enhanced resistance to *Pst* DC3000 (**Figure 2**). SA biosynthetic process and SA metabolic process among the GO terms were significantly enriched in the RNA-seq analysis (**Figure 7**). Thus, *TaBI-1.1* positively regulates the SA signal and modulates the SA-mediated immune response. *PR1* expression was slightly increased in the *atbi1-2* mutant following treatment with *Pst* DC3000, and the extent of the increase of *PR1* expression in *atbi1-2* was significantly lower than that in Col-0 (**Figure 2F**). The SA level in *atbi1-2* was also lower than that in Col-0 under the *Pst* DC3000 treatment. These results support the hypothesis that *atbi1-2* increases the susceptibility to *Pst* DC3000 (**Figure 2C**), suggesting that *AtBI-1* may also be involved in SA-mediated resistance to *Pst* DC3000 and that *TaBI-1.1* may be conserved with *Arabidopsis BI-1* and plays a role in the immune response. However, no significant differences in *PR1* expression and SA levels were observed between *atbi1-2* and Col-0 under mock treatment or at 0 dpi (**Figure 2**). Considering the increase in *PR1* expression and SA levels in *TaBI-1.1* transgenic *Arabidopsis*, we suggest that *PR1* is not modulated by *AtBI-1* alone and that many other interactions may compensate for the deficiency caused by the deletion of *AtBI-1* in *atbi1-2* plants. This compensatory mechanism may be the reason why no significant differences were detected between *atbi1-2* and Col-0 plants in the control group. *TaBI-1* is involved in the response to *Pst. TaBI-1.1* expression was intensely induced in wheat in response to the *Fg* treatment, and it enhanced the resistance to *Pst* DC3000 in *Arabidopsis* (**Figure 2**). Thus, *TaBI-1* may exhibit broad-spectrum pathogen resistance.

In plants, SA is synthesized in plastids from chorismate via two routes. One route is mediated by ICS, which is believed to be responsible for >90% of SA synthesized during the activation of the stress response. The other route uses the phenylalanine ammonia-lyase (PAL)-mediated pathway (Kumar, 2014). *Arabidopsis* encodes two ICS enzymes, ICS1 and ICS2. SA production and pathogen resistance are severely compromised in mutants lacking functional ICS1 (Nawrath and Métraux, 1999). EDS1 is not only required for SA biosynthesis but also promotes the cell death program characteristic of the HR. The absence of EDS1 strongly reduces the HR lesion size and ROS accumulation (Rustérucci et al., 2001). In our study, the expression levels of *ICS1*, *EDS1*, and *PR* genes were significantly up-regulated in *TaBI-1.1* transgenic *Arabidopsis* compared with the levels in Col-0 plants in the control group, indicating that *TaBI-1.1* enhanced SA synthesis (**Figure 3**). The SA level in *TaBI-1.1* transgenic *Arabidopsis* was significantly higher than in Col-0 after *Pst* DC3000 infection (**Figure 2**). Therefore, we hypothesize that the increase in the SA level may be correlated with *ICS1* and *EDS1* expression due to the regulation by *TaBI-1.1*.

Several additional signals directly or indirectly interplay with SA in defense response. SA level are closely connected to ROS production which is involved both upstream and downstream SA signals in response to stress (Herreravásquez et al., 2015). However, an ambivalent effect of SA in promoting ROS accumulation and ROS scavenging has been found in several stress models (Mou et al., 2003; Mateo et al., 2006; Herreravásquez et al., 2015). During early events of signals, SA promotes ROS accumulation which is an essential signal for defense response (Garretón et al., 2002; Lee et al., 2010). Nevertheless, high concentration of exogenous SA exacerbates ROS accumulation and even causes PCD by enhancing H_2_O_2_ production and oxidative damage (Rao and Davis, 1999). Overexpressing *AtBI-1* in tobacco BY-2 cells suppresses H_2_O_2_- or SA-mediated cell death (Kawai-Yamada et al., 2004). Human BI-1 modulates ER stress-induced ROS accumulation (Hyung-Ryong et al., 2009). TaBI-1.1 is an ER-resident protein (**Figure 8**), and constitutive expression of *TaBI-1.1* exhibited an alleviation of damage induced by high concentration of SA in *Arabidopsis* (**Figure 4**). Therefore, *TaBI-1.1* may enhance the tolerance to oxidative stress induced by high concentration of SA on the ER membrane.

Pathogenic infections can trigger a HR in plants, including the production of an oxidative burst, and thereby the rapid PCD in the region surrounding the infection. The suppression of *TaBI-1* transcripts could partially provoke cell death during the wheat stripe rust interaction (Wang et al., 2012). Barley BI-1 functions in regulating cell death during interactions with different kinds of plant pathogens (Eichmann et al., 2004, 2010; Babaeizad et al., 2009). BI-1 as a conserved cell death regulator appears very early in the evolution of eukaryotes and expresses in various organisms. *TaBI-1.1* was intensively induced by *Fg* treatment (Supplementary Table S1), suggesting a possible role of *TaBI-1.1* in the cell death regulation during the interaction with *Fg.* The transcripts of *TaBI-1.1* was detected in various wheat tissues (**Figure 1I**), indicating that it functions as a basal regulator in plants. This finding is in consistent with the ubiquitous expression of BI-1 genes in previous reports (Wang et al., 2012).

Forty-eight up-regulated genes were identified in the RNA-seq analysis (Supplementary Figure S1). The GO terms for the up-regulated genes were mostly enriched in immune response-related processes (**Figure 6**), and the KEGG analysis was enriched in the plant-pathogen interaction and the biosynthesis of secondary metabolite pathways (**Figure 7**). The up-regulated genes exhibited a greater fold enrichment than down-regulated genes, and the results of the GO and KEGG analyses support the phenotype of *TaBI-1.1* in disease resistance (**Figure 2**), indicating that *TaBI-1.1* probably enhances resistance to *Pst* DC3000 by up-regulating gene expression. Seven up-regulated genes related to biotic or abiotic stresses were selected to test the accuracy of the RNA-seq analysis using qRT-PCR. The *HR4* gene is related to bacterial interaction and the hormone response (Saenz-Mata and Jimenez-Bremont, 2012). LOX2 is involved in the JA-regulated defense pathway and suppresses the expression of *ABA1* (Pineda et al., 2012). LURP1 is required for defense against *Hyaloperonospora parasitica*, and the resistance to this pathogen is mediated by the R-proteins RPP4 and RPP5 (Knoth and Eulgem, 2008). *Arabidopsis* plants overexpressing *AtPCR1* exhibit increased Cd resistance, and the reduced *AtPCR1* expression was more sensitive to Cd (Song et al., 2004). *PRX34* knockdown lines exhibit diminished activation of Flg22-activated genes after Flg22 treatment, and the diminished expression of *PRX34* reduced ROS and callose deposition in response to microbe-associated molecular patterns (MAMPs) (Daudi et al., 2012). WAK1, a receptor for OGs (oligogalacturonides), exerts a positive effect on the OG-triggered expression of defense genes and the production of an oxidative burst (Gramegna et al., 2016). Higher expression levels of these genes were detected in *TaBI-1.1* transgenic *Arabidopsis*, and the fold changes were almost consistent with the results of the RNA-seq analysis, suggesting that *TaBI-1.1* probably plays an important role in responses to biotic and abiotic stresses by up-regulating the expression of these genes. However, down-regulated genes were significantly enriched in hormone regulation processes rather than responses to biotic stresses. *GASA4* plays a role in promoting the gibberellic acid (GA) response and the regulation of redox activity, and is up-regulated by GA in dividing cells (Aubert et al., 1998; Rubinovich and Weiss, 2010). *IAA19* is rapidly induced by indole acetic acid (IAA) or brassinolide (BL), partially via the activation of the auxin response element (Nakamura et al., 2004). BZR1, a key transcription factor involved in brassinosteroid (BR) signaling, directly binds to the promoters of *IAA19* and *ARF7*, which is helpful to establish and maintain the morphology of *Arabidopsis* seedlings grown in the dark (Zhou et al., 2013). In addition, the *PIP3* (*PIP2;7*) gene, which is down-regulated in *TaBI-1.1* transgenic *Arabidopsis*, is involved in negatively regulating salt stress (Pou et al., 2016), suggesting that *TaBI-1.1* transgenic *Arabidopsis* has the potential to exhibit enhanced salt tolerance.

Abscisic acid is the central regulator in abiotic stress response and coordinates an array of functions to enable plants to cope with various stresses (Sah et al., 2016). In this work, *TaBI-1.1* expression was up-regulated by SA and down-regulated by ABA (**Figure 1**). During the germination and seedling stages, *TaBI-1.1* transgenic *Arabidopsis* exhibited decreased sensitivity to ABA, and *atbi1-2* showed the opposite phenotype as transgenic plants (**Figure 4**), indicating that both *TaBI-1.1* and *AtBI-1* functions negatively in ABA signals. The expression levels of *CaBI-1* and *TaBI-1* have been reported to be up-regulated by exogenous ABA (Wang et al., 2012). Clearly, the expression of *TaBI-1.1* observed in response to the ABA treatment differs from that of *CaBI-1* and *TaBI-1*. The possible reasons may be that there are some different expression patterns for *BI-1* under ABA treatment in different plant species, and different growth stages and ABA concentrations have different impacts on *BI-1* expression. *CaBI-1* expression in leaves is up-regulated by high salinity, similar to the results for *TaBI-1.1*. Thus, BI-1, a conserved cytoprotective protein in plants, may exhibit a variety of functions in different species grown under abiotic stresses.

AtBI-1 modulates ER stress-mediated PCD (Watanabe and Lam, 2008). The loss of function of *Ss-Bi1* increases sensitivity to heat stress and ER stress (Yu et al., 2015). Human BI-1 regulates ER stress-associated ROS accumulation (Hyung-Ryong et al., 2009). BI-1, an ER-resident transmembrane protein, is highly conserved in plants and mammals. As AtBI-1 and TaBI-1.1 are both localized to the ER membrane, we hypothesize that TaBI-1.1 may also be involved in the response to ER stress (**Figure 8**). TaPIP1, a protein that interacts with TaBI-1.1, is also localized to the ER membrane. More importantly, the two proteins are co-localized to the ER membrane, indicating that the interaction between TaBI-1.1 and TaPIP1 may occur in the ER membrane. PIP is a subgroup of aquaporins (AQPs) that is conserved across numerous plant species (Kaldenhoff and Fischer, 2006). Based on accumulating evidence, *AQP* responds to various stresses. For instance, overexpression of *TaAQP8* in tobacco increases tolerance to salt stress by retaining a high K^+^/Na^+^ ratio and high Ca^2+^ content and enhances the antioxidant system to reduce H_2_O_2_ accumulation and membrane damage (Hu et al., 2012). Transgenic rice plants overexpressing an aquaporin *RWC3* (*OsPIP1;3*) exhibit higher root osmotic hydraulic conductivity (Lp), leaf water potential and relative cumulative transpiration in response to a polyethylene glycol (PEG) treatment (Lian et al., 2004). The ortholog of TaPIP1 in *Arabidopsis*, AtPIP1;4, was shown to be involved in responses to both abiotic and biotic stresses. Overexpression of *AtPIP1;4* enhances water flow and facilitates germination in response to cold stress (Jang et al., 2007). *TaPIP1* is induced by NaCl, ABA and H_2_O_2_ treatments, and overexpression of *TaPIP1* in transgenic *Arabidopsis* confers salt tolerance during germination stage and up-regulates the expression of a number of stress-associated genes (Han et al., 2014). *TaBI-1.1* was induced by NaCl treatment and down-regulated under ABA treatment, and constitutive expression of *TaBI-1.1* didn’t show significant salt tolerance and decreased the sensitivity to ABA in *Arabidopsis* (**Figures 1**, **4**, **5**), indicating that *TaBI-1.1* and *TaPIP1* may play opposite roles in response to ABA signals and exhibit different functions under salt tolerance. *AtPIP1;4* mediates the translocation of externally applied H_2_O_2_ from the apoplast to the cytoplasm, which is required for the cytoplasmic import of pathogen-associated molecular pattern (PAMP)-induced apoplastic H_2_O_2_, indicating its pivotal role in apo-cytoplastic signal transduction in immunity pathways (Tian et al., 2016). ROS and SA could act synergistically in a signal amplification loop in defense response. In this study, both *TaBI-1.1* and *TaPIP1* expression levels were up-regulated under SA treatment and enhanced the resistance to *Pst* DC3000 infection (**Figures 1**, **2**, **8D**, **9**), implying *TaBI-1.1* and *TaPIP1* exhibited similar roles in response to pathogen infection. Based on these results, we hypothesize that TaPIP1 as an ER membrane channel protein may function in the translocation of ROS, and TaBI-1.1 mediates the signal amplification loop of ROS and SA by interacting with TaPIP1 in defense response. Nevertheless, the mechanism of the interaction in biological processes are unable to be determined, and additional investigations are required.

## Author Contributions

Z-SX coordinated the project, conceived and designed the experiments, and edited the manuscript. P-PL performed the experiments and wrote the first draft. T-FY conducted the bioinformatic work and performed the experiments. W-JZ revised and edited the manuscript. MC and Y-JX contributed with valuable discussions. JC and Y-BZ provided the analytical tools and managed the reagents. Y-ZM coordinated the project. All authors have read and approved the final manuscript.

## Conflict of Interest Statement

The authors declare that the research was conducted in the absence of any commercial or financial relationships that could be construed as a potential conflict of interest.

## Footnotes

1 http://tophat.cbcb.umd.edu/
