# Supplementary figures and images for: The Wheat Bax Inhibitor-1 Protein Interacts with an Aquaporin TaPIP1 and Enhances Disease Resistance in Arabidopsis
Source: Front Plant Sci. 2018 Jan 22;9:20. doi: 10.3389/fpls.2018.00020 (PMC5786567; doi:10.3389/fpls.2018.00020)

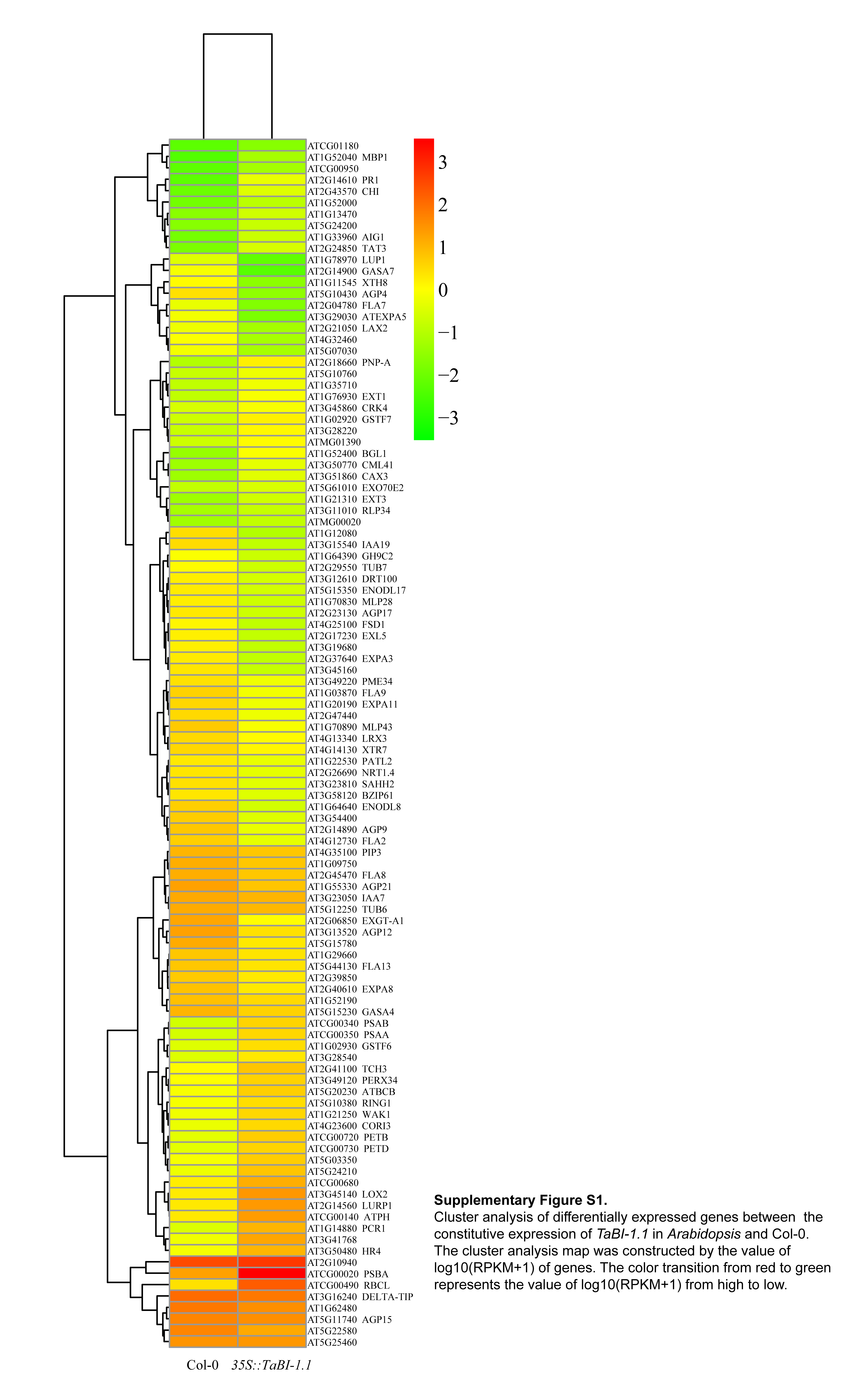

Supplement: Supplementary file 3 [file Image_1.tif]
